# Biochemical Recurrence Surrogacy for Clinical Outcomes After Radiotherapy for Adenocarcinoma of the Prostate

**DOI:** 10.1200/JCO.23.00617

**Published:** 2023-08-28

**Authors:** Soumyajit Roy, Tahmineh Romero, Jeff M. Michalski, Felix Y. Feng, Jason A. Efstathiou, Colleen A.F. Lawton, Michel Bolla, Philippe Maingon, Theo de Reijke, David Joseph, Wee Loon Ong, Matthew R. Sydes, David P. Dearnaley, Alison C. Tree, Nathalie Carrier, Abdenour Nabid, Luis Souhami, Luca Incrocci, Wilma D. Heemsbergen, Floris J. Pos, Almudena Zapatero, Araceli Guerrero, Ana Alvarez, Carmen Gonzalez San-Segundo, Xavier Maldonado, Robert E. Reiter, Matthew B. Rettig, Nicholas G. Nickols, Michael L. Steinberg, Luca F. Valle, T. Martin Ma, Matthew J. Farrell, Beth K. Neilsen, Jesus E. Juarez, Jie Deng, Sitaram Vangala, Norbert Avril, Angela Y. Jia, Nicholas G. Zaorsky, Yilun Sun, Daniel Spratt, Amar U. Kishan

**Affiliations:** ^1^ Department of Radiation Oncology, Rush University Medical Center, Chicago, IL; ^2^ Department of Medicine Statistics Core, David Geffen School of Medicine, University of California Los Angeles, Los Angeles, CA; ^3^ Department of Radiation Oncology, Washington University, St Louis, MO; ^4^ Department of Radiation Oncology, University of California San Francisco, San Francisco, CA; ^5^ Department of Radiation Oncology, Massachusetts General Hospital, Harvard Medical School, Boston, MA; ^6^ Department of Radiation Oncology, Medical College of Wisconsin, Milwaukee, WI; ^7^ Radiotherapy Department, University Hospital, Grenoble, France; ^8^ Department of Oncology, Hematology, and Supportive Care, Sorbonne University, Paris, France; ^9^ Department of Urology, Prostate Cancer Network in the Netherlands, Amsterdam University Medical Centers, University of Amsterdam, Amsterdam, the Netherlands; ^10^ Department of Medicine and Surgery, University of Western Australia, Perth, WA, Australia; ^11^ Alfred Health Radiation Oncology, Monash University, Melbourne, VIC, Australia; ^12^ MRC Clinical Trials Unit at UCL, Institute of Clinical Trials and Methodology, University College London, London, United Kingdom; ^13^ Division of Radiotherapy and Imaging, The Institute of Cancer Research and Department of Urology, The Royal Marsden NHS Foundation Trust, London, United Kingdom; ^14^ Department of Radiation Oncology, University of Washington, Seattle, WA; ^15^ Clinical Research Center, Centre Hospitalier Universitaire de Sherbrooke, Sherbrooke, QC, Canada; ^16^ Department of Radiation Oncology, Centre Hospitalier Universitaire de Sherbrooke, Sherbrooke, QC, Canada; ^17^ Department of Radiation Oncology, McGill University Health Centre, Montréal, QC, Canada; ^18^ Department of Radiation Oncology, Erasmus MC Cancer Institute, Rotterdam, the Netherlands; ^19^ Department of Radiation Oncology, Netherlands Cancer Institute-Antoni van Leeuwenhoek Hospital, Amsterdam, the Netherlands; ^20^ Hospital Universitario de la Princesa, Madrid, Spain; ^21^ Hospital Son Espases, Palma de Mallorca, Spain; ^22^ Hospital General Universitario Gregorio Marañón, Madrid, Spain; ^23^ Hospital Universitari Vall d'Hebron, Barcelona, Spain; ^24^ Department of Urology, University of California Los Angeles, Los Angeles, CA; ^25^ Department of Medical Oncology, University of California Los Angeles, Los Angeles, CA; ^26^ Department of Radiation Oncology, University of California Los Angeles, Los Angeles, CA; ^27^ Department of Radiology, Division of Nuclear Medicine, Case Comprehensive Cancer Center, Case Western Reserve University, Cleveland, OH; ^28^ Department of Radiation Oncology, University Hospitals Seidman Cancer Center, Case Western Reserve University School of Medicine, Cleveland, OH; ^29^ Department of Population Quantitative Health Sciences, Case Western Reserve University, Cleveland, OH

## Abstract

**PURPOSE:**

The surrogacy of biochemical recurrence (BCR) for overall survival (OS) in localized prostate cancer remains controversial. Herein, we evaluate the surrogacy of BCR using different surrogacy analytic methods.

**MATERIALS AND METHODS:**

Individual patient data from 11 trials evaluating radiotherapy dose escalation, androgen deprivation therapy (ADT) use, and ADT prolongation were obtained. Surrogate candidacy was assessed using the Prentice criteria (including landmark analyses) and the two-stage meta-analytic approach (estimating Kendall's tau and the *R*^2^). Biochemical recurrence-free survival (BCRFS, time from random assignment to BCR or any death) and time to BCR (TTBCR, time from random assignment to BCR or cancer-specific deaths censoring for noncancer-related deaths) were assessed.

**RESULTS:**

Overall, 10,741 patients were included. Dose escalation, addition of short-term ADT, and prolongation of ADT duration significantly improved BCR (hazard ratio [HR], 0.71 [95% CI, 0.63 to 0.79]; HR, 0.53 [95% CI, 0.48 to 0.59]; and HR, 0.54 [95% CI, 0.48 to 0.61], respectively). Adding short-term ADT (HR, 0.91 [95% CI, 0.84 to 0.99]) and prolonging ADT (HR, 0.86 [95% CI, 0.78 to 0.94]) significantly improved OS, whereas dose escalation did not (HR, 0.98 [95% CI, 0.87 to 1.11]). BCR at 48 months was associated with inferior OS in all three groups (HR, 2.46 [95% CI, 2.08 to 2.92]; HR, 1.51 [95% CI, 1.35 to 1.70]; and HR, 2.31 [95% CI, 2.04 to 2.61], respectively). However, after adjusting for BCR at 48 months, there was no significant treatment effect on OS (HR, 1.10 [95% CI, 0.96 to 1.27]; HR, 0.96 [95% CI, 0.87 to 1.06] and 1.00 [95% CI, 0.90 to 1.12], respectively). The patient-level correlation (Kendall's tau) for BCRFS and OS ranged between 0.59 and 0.69, and that for TTBCR and OS ranged between 0.23 and 0.41. The *R*^2^ values for trial-level correlation of the treatment effect on BCRFS and TTBCR with that on OS were 0.563 and 0.160, respectively.

**CONCLUSION:**

BCRFS and TTBCR are prognostic but failed to satisfy all surrogacy criteria. Strength of correlation was greater when noncancer-related deaths were considered events.

## INTRODUCTION

Because of the long natural history of prostate cancer (PCa), clinical trials that investigate management strategies in localized PCa need prolonged follow-up and large sample sizes to show overall survival (OS) benefits. Thus, significant efforts have been invested in exploring the utility of surrogate end points. One such candidate end point is biochemical recurrence (BCR), which is a prostate-specific antigen (PSA)–based end point that occurs much earlier in the natural history.^1^ Two meta-analyses have shown that event-free survival, a composite PSA-based end point, failed to meet surrogacy criteria for OS. The first used the two-stage meta-analytic approach with individual patient data (IPD),^2-4^ and the second used the second condition of the two-stage approach with trial-level data.^5^ Both demonstrated that current PSA-based end points were not able to serve as surrogate end points for randomized trials. By contrast, a secondary analysis of Radiation Therapy Oncology Group (RTOG) 9202 showed that the time interval to BCR met the Prentice criteria for surrogacy.^6,7^

**CONTEXT**

**Key Objective**
Biochemical recurrence (BCR) is an early event in the natural history of prostate cancer (PCa), and it is therefore of great interest as a potential surrogate end point for overall survival (OS). Previous surrogacy analyses have yielded conflicting conclusions about the surrogacy potential of BCR. Herein, we performed an individual patient data meta-analysis of 11 randomized controlled trials that used treatment intensification strategies with definitive radiotherapy known to reduce BCR to evaluate its surrogacy. We used both the Prentice criteria and the two-stage meta-analytic approach to evaluate the surrogacy of BCR-free survival (other-cause mortality as an event) and time to BCR (other-cause mortality as a competing risk).
**Knowledge Generated**
Overall, there was a poor to modest correlation between these BCR-based surrogate end points and OS, suggesting that BCR-based end points are not appropriate surrogate end points. The strength of its surrogacy potential appears to be related to the censoring mechanism used, and in addition, the magnitude of improvement in BCR-based end points to suggest an ultimate nonzero effect on OS varies between intensification strategies.
**Relevance *(M.A. Carducci)***
Despite attempt to show potential surrogacy for OS using BCR as a potential marker, these results strongly suggest that BCR-based endpoints should not be the primary endpoint in randomized trials conducted for localized PCa. Metastasis-free survival remains an appropriate endpoint for prospective trials related to radiation therapy in localized disease.*

*Relevance section written by *JCO* Associate Editor Michael A. Carducci, MD, FACP, FASCO.

In addition to methodologic differences in surrogacy criteria, the conflicting results could also be explained by the difference in definition of censoring and events between the two studies. In the ICECaP meta-analysis, patients with death without previous recurrence (ie, who experienced other-cause mortality) were censored at the time of last PSA assessment if the interval between the last PSA assessment and death was >15 months. However, in the secondary analysis of RTOG 9202, death of any cause was considered an event. The handling of other-cause mortality as a censoring event versus an end point event is pivotal as this could influence the surrogacy potential of a BCR-based end point given the high risk of competing mortality. To further investigate the impact of the specific surrogate criteria and censoring mechanism chosen on the surrogacy candidacy of BCR, we performed an IPD meta-analysis of patients with localized PCa from 11 randomized controlled trials from the Meta-Analysis of Randomized trials in Cancer of the Prostate (MARCAP) consortium, using both the Prentice criteria and the two-stage meta-analytic approach and evaluating both biochemical recurrence-free survival (BCRFS; other-cause mortality as an event) and time to BCR (TTBCR; other-cause mortality as a competing risk) as candidate surrogate end points.

## MATERIALS AND METHODS

This IPD meta-analysis was performed using trial data from the MARCAP consortium, which has been described previously.^8^ Briefly, it contains IPD from randomized clinical trials run through multiple collaborative groups including the European Organization for Research and Treatment of Cancer (EORTC), RTOG (now National Surgical Adjuvant Breast and Bowel Project, Radiation Therapy and Oncology Group, and Gynecologic Oncology Group [NRG] Oncology; NRG/RTOG), Medical Research Council (MRC), Institute of Cancer Research, Prostate Cancer Study group (PCS), the Grupo de Investigación Clínica en Oncología Radioterápica (GICOR), and the Ottawa Hospital Research Institute.

### Trial Selection

As our goal was to evaluate the surrogacy potential of BCR-based end points, we chose to focus on interventions that have been tested in multiple randomized trials and been shown to improve BCR-based outcomes. This led to focusing on three forms of intensification: radiation therapy (RT) dose escalation, the addition of short-term androgen deprivation therapy (ADT), and the prolongation of short-term ADT to long-term ADT. We included 11 randomized controlled trials that investigated treatment intensification using (1) RT dose escalation (MRC RT01, CKTO 9610, PCS III, and RTOG 0126), (2) addition of ADT to RT (RTOG 9408, PCS III, EORTC 22991, Trans-Tasman Radiation Oncology Group [TROG] 96.01), and (3) prolongation of short-term ADT to long-term ADT (RTOG 9202, EORTC 22961, DART/GICOR 01/05, and TROG Randomised Androgen Deprivation and Radiotherapy [RADAR]), respectively.^9-18^ The PCS III trial contributed patients to trial groups 1 and 2. Details regarding individual trials are given in the Data Supplement (Table A1, online only).

### Statistical Considerations

The primary objective of this study was to determine if TTBCR is a surrogate intermediate clinical end point (ICE) for OS in patients with localized PCa treated with RT with or without ADT. In addition, we also investigated if changing the underlying censoring mechanism causes any differential impact on the surrogacy results by evaluating the surrogacy of BCRFS. We applied both the Prentice criteria and the two-stage meta-analytic approach to evaluate surrogacy of BCR for OS, which was defined as the time from random assignment to death from any cause or date of last follow-up.^7,19^

The Prentice criteria include the following: (1) treatment should have significant effect on the true end point and (2) the ICE; (3) there is a significant association between the ICE and the true end point; and (4) the effect of the treatment on the true end point is mediated by the effect of treatment on the ICE.^7^ To investigate the first two criteria, we evaluated the treatment effect on BCR and OS by computing the cause-specific hazard ratio (HR) in the three different groups of intensification trials, whereas for the third and fourth criteria, we used landmark analyses. For determining the cause-specific HR of BCR, BCR (as defined in individual trials, Data Supplement, Table A2) was considered as events, whereas patients without BCR were censored. HRs were estimated using multilevel multivariable Cox proportional hazard models. The models included other prognostic covariates because of their prognostic association as reported in the previous literature to adjust for any imbalance induced by defining the relevant cohorts for the landmark analyses.^20^ Additional details are provided in the Data Supplement.

The two-stage meta-analytic approach to evaluating surrogacy is based on two conditions.^19^ Condition 1 requires the surrogate and the true end point to be correlated, whereas condition 2 requires the treatment effects on the surrogate and the true end points to be correlated. Here, we evaluated BCRFS and TTBCR separately. BCRFS was defined as the time from random assignment to biochemical or clinical recurrence or the start of salvage ADT in the absence of recurrence or deaths from any cause, with censoring if patients were lost to follow-up or survived to the end of the follow-up period. TTBCR was defined as the time from random assignment to biochemical or clinical recurrence or the start of salvage ADT in the absence of recurrence or cancer-specific deaths, with patients censored if they were lost to follow-up or died of other causes or survived to the end of the follow-up period. OS was defined as the time from random assignment to death. The validity of the surrogate is reflected by the strength of both correlations. Condition 1 was examined at both the patient and trial levels. At the patient level, we applied a bivariate Copula model, modeling both the time to surrogate end point and true end point to evaluate Kendall's τ, measuring the rank correlation between the end points, with bootstrapped 95% confidence intervals (CIs). At the trial level, the correlation of the treatment effect on the surrogate and true end point was determined using a weighted linear regression model (additional details are provided in the Data Supplement). To be consistent with previous work and other surrogacy assessments in oncology, we defined the a priori threshold of clinically relevant surrogacy as *R*^2^ ≥ 0.7 and Kendall's tau ≥0.7.^4,21^ All analyses were performed using SAS 15.1 by SAS Institute Inc, Cary, NC, and R version 4.1.0 (2021-05-18, The R Foundation for Statistical Computing, Vienna, Austria) with its packages for statistical analysis.

## RESULTS

A total of 10,741 patients from 11 randomized trials were included. Overall, 3,639 were treated on trials evaluating RT dose escalation, 3,930 on trials evaluating ADT use, and 3,772 on trials of ADT prolongation (Data Supplement, Table A3). Individual trial treatment effects and funnel plots for all end points of interest are shown in the Data Supplement. The median follow-up was 9.2 years (IQR, 6.1-11.5) overall and was 8.8 years (IQR, 6.7-10.5) for the RT dose-escalation group, 10.6 years (IQR, 6.6-13.4) for the ADT use group, and 8.6 years (IQR, 5.6-11.2) for the ADT prolongation group. In the overall cohort, the median age was 70 years (IQR, 65-74); 73% had cT1/T2 disease, 85% had Gleason ≤7 disease, and the median baseline PSA was 11.1 ng/mL (IQR, 7.1-18.0).

The results for multivariable Cox regression for treatment effects on BCR and OS are shown in Table 1. In trials that investigated the utility of RT dose escalation, there was a 29% reduction in the hazard of BCR with dose-escalated RT (HR, 0.71; 95% CI, 0.63 to 0.79). However, RT dose escalation had no significant effect on OS (HR, 0.98; 95% CI, 0.87 to 1.11), failing to meet the second Prentice criterion. Addition of short-term ADT to RT was associated with a significant reduction in the hazard of BCR (HR, 0.53; 95% CI, 0.48 to 0.59) and death (HR, 0.91; 95% CI, 0.84 to 0.99). Prolongation of ADT was associated with a significant reduction in the hazard of BCR (HR, 0.54; 95% CI, 0.48 to 0.61) and death (HR, 0.86; 95% CI, 0.78 to 0.94).

**TABLE 1.**
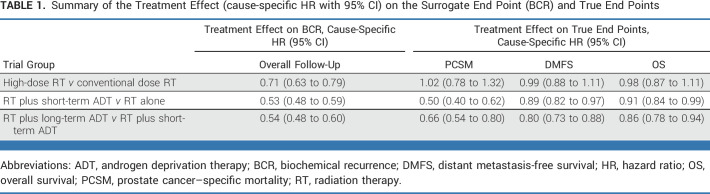
Summary of the Treatment Effect (cause-specific HR with 95% CI) on the Surrogate End Point (BCR) and True End Points

The results of landmark analyses are shown in Table 2, which shows that the third Prentice criterion was satisfied for all three groups of trials. In the first set of trials (low- *v* high-dose RT), BCR events by 48 months (HR, 2.46 [95% CI, 2.08 to 2.92]) after random assignment were associated with significantly increased hazard of deaths. Similar findings were seen in the second set of trials (RT alone *v* RT plus ADT) where BCR events by 48 months (HR, 1.51 [95% CI, 1.35 to 1.70]) after random assignment were associated with significantly inferior OS. Finally, in the third set of trials comparing effects of long-term versus short-term ADT with RT, BCR events by 48 months (HR, 2.31 [95% CI, 2.04 to 2.61]) were also associated with significantly inferior OS. Similar associations were found between BCR and distant metastasis-free survival (DMFS) and prostate cancer–specific mortality (PCSM) for all three groups of trials, respectively **(**Data Supplement, Table A4).

**TABLE 2.**
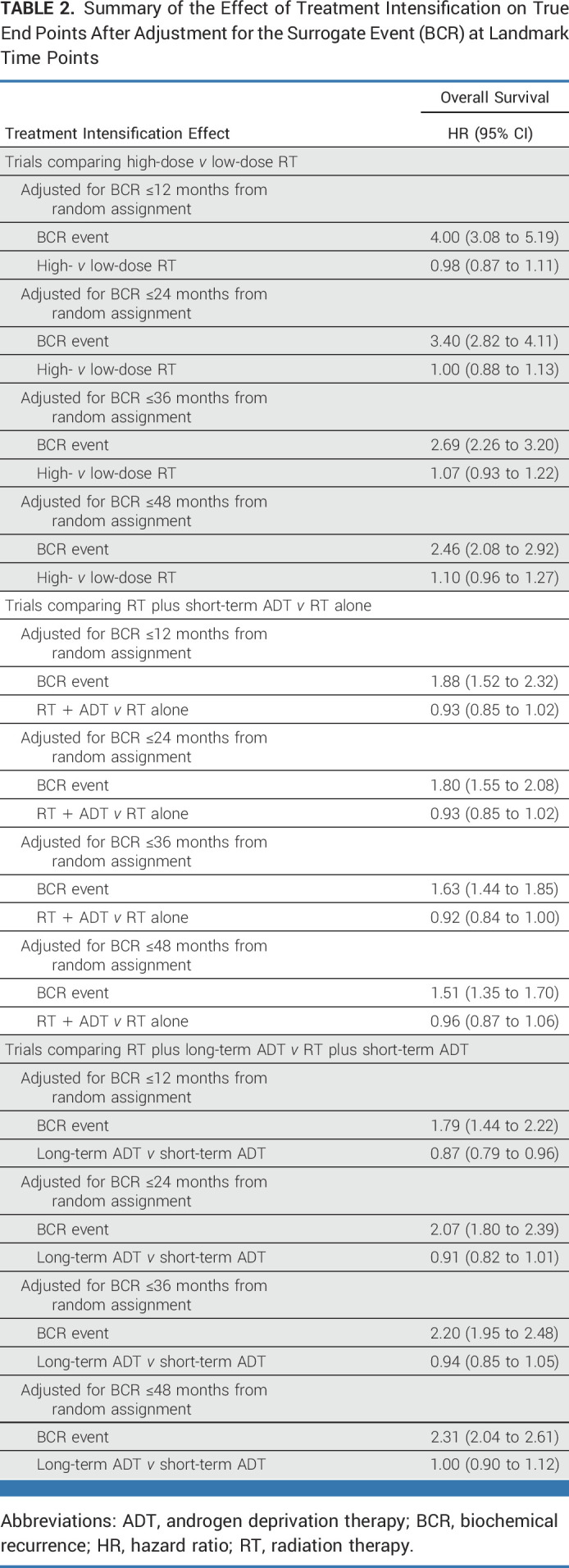
Summary of the Effect of Treatment Intensification on True End Points After Adjustment for the Surrogate Event (BCR) at Landmark Time Points

After adjusting for BCR at landmark time points, we did not find any consistent treatment effect from RT dose escalation on OS (Table 2). For trials investigating the effect of short-term ADT to RT, there was a small and nonsignificant evidence of beneficial treatment effects from the combination of RT plus short-term ADT on OS after adjustment for BCR by 48 months after random assignment (HR, 0.96 [95% CI, 0.87 to 1.06]). Finally, in trials evaluating the benefit of prolongation of ADT duration with RT, there was some evidence of beneficial treatment effects from ADT prolongation on OS after adjustment for BCR by 36 months (HR, 0.94 [95% CI, 0.85 to 1.05]) after random assignment, whereas no effect was seen at 48 months (HR, 1.00 [95% CI, 0.90 to 1.12]). Overall, the fourth Prentice criterion was not met consistently across the three trial groups. The treatment effects on DMFS and PCSM after adjustment for BCR at the landmark time points are summarized in the Data Supplement (Table A4)**.**

### Meta-Analytic Approach

The patient-level correlation between TTBCR (non-PCa deaths censored) and OS was low with Kendall's τ values of 0.371 (95% CI, 0.364 to 0.373), 0.233 (95% CI, 0.227 to 0.235), and 0.414 (95% CI, 0.408 to 0.415), respectively, in the three groups of trials. However, the correlation between BCRFS and OS ranged from low to moderate. In the three groups of trials, Kendall's τ for patient-level correlation between BCRFS and OS was 0.693 (95% CI, 0.690 to 0.694), 0.589 (95% CI, 0.587 to 0.591), and 0.651 (95% CI, 0.648 to 0.652), respectively. At the trial level, trial-specific treatment effects on surrogate and true end points, measured by log HR for each end point, are shown in forest plots in the Data Supplement (Figs A1-A9).

**FIG 1.**
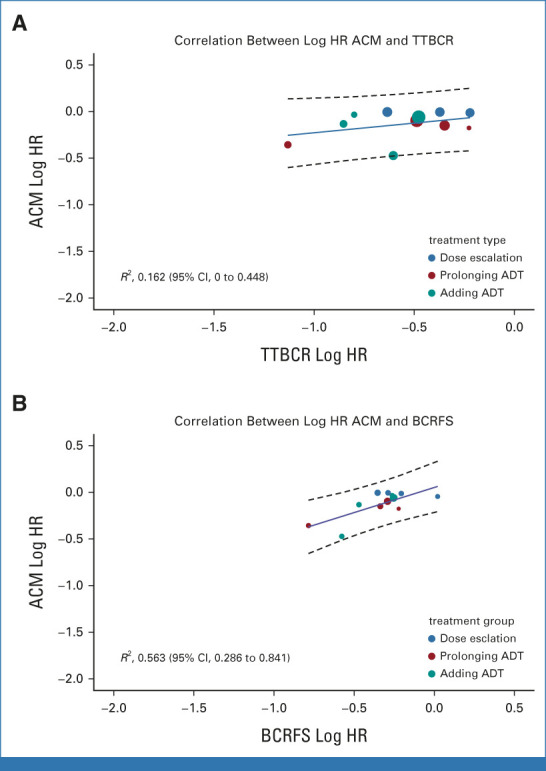
Bubble plot and weighted linear regression for the treatment effect (log HR) for ACM and the treatment effect (log HR) for (A) TTBCR and (B) BCRFS. Circle size and regression were weighted by inverse variance of log hazard estimates for surrogate events. BCRFS was defined as the time from random assignment to biochemical or clinical recurrence or the start of salvage ADT in the absence of recurrence or deaths from any cause, with censoring if patients were lost to follow-up or survived to the end of the follow-up period. TTBCR was defined as the time from random assignment to biochemical or clinical recurrence or the start of salvage ADT in the absence of recurrence or cancer-specific deaths, with patients censored if they were lost to follow-up or died of other causes or survived to the end of the follow-up period. ACM, all-cause mortality; ADT, androgen deprivation therapy; BCRFS, biochemical recurrence-free survival; HR, hazard ratio; TTBCR, time to BCR.

**FIG 2.**
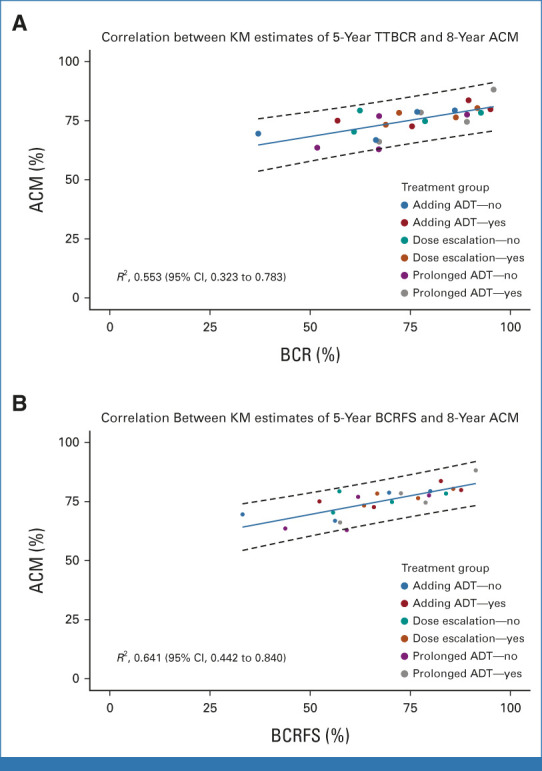
Bubble plot and regression of ACM at 8 years and (A) TTBCR and (B) BCRFS at 5 years. All estimates are Kaplan-Meier estimates by trial and treatment arm. BCRFS was defined as the time from random assignment to biochemical or clinical recurrence or the start of salvage ADT in the absence of recurrence or deaths from any cause, with censoring if patients were lost to follow-up or survived to the end of the follow-up period. TTBCR was defined as the time from random assignment to biochemical or clinical recurrence or the start of salvage ADT in the absence of recurrence or cancer-specific deaths, with patients censored if they were lost to follow-up or died of other causes or survived to the end of the follow-up period. ACM, all-cause mortality; ADT, androgen deprivation therapy; BCRFS, biochemical recurrence-free survival; KM, Kaplan-Meier; TTBCR, time to BCR.

The *R*^2^ value from the weighted linear regression of log HR for OS and log HR for TTBCR was 0.162 (95% CI, 0 to 0.448; Fig 1A). For BCRFS, the *R*^2^ value from the weighted linear regression of log HR for OS and log HR for the surrogate event was 0.563 (95% CI, 0.286 to 0.841; Fig 1B). The *R*^2^ value between the Kaplan-Meier estimate of 5-year TTBCR and 8-year OS was 0.553 (95% CI, 0.323 to 0.783; Fig 2A), whereas that between 5-year BCRFS and 8-year OS was 0.641 (95% CI, 0.442 to 0.840; Fig 2B). None of these *R*^2^ values met the predefined threshold of strong trial-level correlation of the treatment effect on surrogate and true end points. We estimated the surrogate threshold effect (STE), defined as the maximum value of the HR for BCRFS (HR_BCRFS_) or TTBCR (HR_TTBCR_) that needed to be observed in a trial to ensure the possibility of a nonzero effect on OS. The STE in terms of maximum HR_TTBCR_ could not be determined for RT dose escalation and ADT use trials. The STE in terms of maximum HR_TTBCR_ was 0.20 for ADT prolongation trials. Similarly, for RT dose-escalation trials, STE in terms of maximum HR_BCRFS_ could not be determined. However, for ADT use trials and ADT prolongation trials, the STEs in terms of maximum HR_BCRFS_ were 0.69 and 0.29, respectively.

## DISCUSSION

In this meta-analysis, we applied both Prentice criteria and the two-stage meta-analytic approach to determine the surrogacy of BCR for OS in men with localized PCa who received RT with or without ADT. Overall, we found that although BCR was associated with increased risk of deaths, it did not meet the criteria to be a surrogate for OS. One of the potential reasons for this lack of surrogacy could be the preponderance of non–cancer-specific deaths in this patient population,^22^ which was reflected in the varying strength of patient-level and trial-level correlations between the surrogate end point and OS depending on the censoring of noncancer-related deaths. Most notably, Kendall's τ at the patient level for correlation between BCRFS and OS ranged from 0.59 to 0.69, whereas for TTBCR, Kendall's τ values ranged from 0.23 to 0.41. Similarly, the *R*^2^ value for the correlation between log HR of OS and log HR of BCRFS was 0.56, whereas it was only 0.16 for the correlation between log HR of OS and log HR of TTBCR.

Our findings help clarify the conflicting results reported by the ICECaP group, which used the two-stage meta-analytic approach to determine surrogacy of a composite PSA-based end point (of which approximately 2/3 of events were BCR), and the findings of Dignam et al who used the Prentice criteria to demonstrate surrogacy between the time interval to BCR and OS.^4,6^ Our findings largely agree with the conclusions of the ICECaP group (which included seven of the trials evaluated in the present analysis) and a second, trial-level meta-analysis,^5^ both of which found that a largely PSA-based end point was not a valid surrogate for OS. The present analysis adds to these by using the two-stage meta-analytic approach and analyzing the Prentice criteria for the surrogacy of BCR specifically, using IPD and harmonizing the investigated intensification strategies of the trials. On the basis of the present meta-analysis, ADT prolongation should reduce the hazard of BCR or death by at least 71% to have a nonzero effect on OS, whereas addition of ADT to RT should reduce the hazard of BCR or death by at least 31% to have a nonzero effect on OS. However, previous IPD analyses from the MARCAP group suggest that the upper bounds of the 95% CI for BCRFS is 0.95 for pure dose escalation, 0.79 for adding short-term ADT to low-dose RT, and 0.64 for adding short-term ADT to high-dose RT, suggesting that BCRFS would not be a reliable surrogate for OS in the context of these interventions.^23^ In fact, in the context of dose escalation, there was a slight inverse correlation between BCRFS and TTBCR and OS. Although the upper bounds of the 95% CI for BCRFS for prolonging ADT were 0.52 in the context of low-dose RT and 0.46 in the context of high-dose RT,^23^ we were not able to identify a STE for this intervention in our present analysis. Thus, while BCRFS may, in theory, function as a surrogate in certain contexts, it does not reliably do so across these rigorously studied intensification strategies. The observed differences in patient-level correlation of BCRFS (or TTBCR) with OS between the three groups of trials may be affected by differential durations of gonadal suppression and post-testosterone recovery subsequent PSA kinetics.^24-26^ When TTBCR was used rather than BCRFS (meaning that other-cause mortality was censored), correlations eroded significantly at both the patient and trial level. Overall, these findings suggest that the strength of association between BCR and OS varies on the basis of the underlying censoring mechanism used to define the BCR-based end point. Thus, while BCR is indeed of important prognostic significance, its use as a surrogate end point should be discouraged. More broadly, the findings underscore the importance of understanding censoring, particularly in situations where other-cause mortality can potentially outweigh cancer-specific mortality.

As with any study, this meta-analysis is not without limitations. First, while the MARCAP consortium was able to provide data for most of the relevant phase III clinical trials of each treatment intensification strategy, the analysis does not include all such trials ever conducted. The definition of BCR varied between trials, ranging from the older ASTRO definition to the more recent Phoenix criteria, which could affect the threshold at which salvage therapy was instituted.^1^ This could potentially bias the correlation between BCR and subsequent longevity. A small proportion of patients had low-risk disease, which might have further downplayed the strength of correlation between BCRFS or TTBCR and OS. Furthermore, the study included trials that were conducted approximately over more than two decades during which patient selection, staging, diagnostic criteria including grade grouping, and therapeutic approaches including RT and ADT have witnessed significant evolution.^27,28^ Analysis of individual trial effects and funnel plots suggest that although intertrial heterogeneity exists, as to be expected, the overall effect sizes were relatively consistent, strengthening the meta-analytic approach. The use of multiparametric magnetic resonance imaging has allowed for higher accuracy of detecting clinically meaningful cancer, better patient selection, and radiation dose escalation.^27,29-31^ Staging investigations evolved from the use of conventional imaging to the use of prostate-specific membrane antigen (PSMA)–based positron emission tomography (PET) scans.^32^ Similar evolution has taken place in post-treatment response assessment. Although PSA has continued to be an integral part of the post-treatment evaluation process, use of PSMA-based PET has ushered a new era by early detection of metastatic disease.^33^ This can potentially alter treatment decisions in several patients in the recurrent setting and might confound the prognostic link between BCR and OS. In addition, although it is shown that BCR is not a surrogate of OS, it still has a significant impact in the evolving disease course of patients with PCa. Patients who experience BCR may receive salvage systemic therapies, particularly if distant metastases develop, and others may pursue local salvage therapies that can cause other toxicities. These salvage therapies might have immense ramifications on the overall quality of life for those patients and their families. Finally, from an oncologic efficacy standpoint, salvage treatment options including systemic therapies have significantly improved for men with recurrent metastatic castrate-sensitive or castrate-resistant PCa.^34-40^ The continuously changing landscape of systemic therapy could have affected our association given the wide spectrum of trials over the past few decades. Further improvement in systemic therapy over time could potentially nullify the strength of correlation of the treatment effect on BCR with that on OS in these patients.

Overall, these results strongly suggest that BCR-based end points should not be the primary end point of any randomized trial in localized PCa. Moreover, when designing confirmatory randomized trials on the basis of phase II readouts of a BCR-based benefit, the power calculation and feasibility analysis must consider that an extremely large effect on a BCR-based end point would be necessary to expect an ultimate OS benefit. It should also be noted that the emergence of prognostic and predictive biomarkers might affect the surrogacy of BCR in different ways. Given that our findings demonstrate the prognostic association between BCR and OS and the relative importance of noncancer-related deaths and cancer-specific deaths in nonmetastatic PCa, BCR is clearly not an ideal end point for biomarker development or validation in general. Nonetheless, it is possible that a biomarker might identify a select group of patients with extremely aggressive disease, and the correlation between BCR-based end points and OS may be stronger in these patients compared with others.^41^ Still, such patients are likely to also receive intensified salvage therapy including life-prolonging systemic therapy that could erode the surrogacy of BCR for OS. Thus, only data from specifically designed prospective trials can provide further clarity on the potential surrogacy of BCR for such patients. At this point, metastasis-free survival is a more appropriate end point for prospective clinical trials investigating strategies related to RT in localized PCa.

In conclusion, in this IPD-based meta-analysis, we noted that BCR is prognostic for OS; however, neither BCRFS nor TTBCR consistently met surrogacy criteria for OS. The variability in the strength of association between the surrogate and true end point with higher strength when BCRFS was used as a surrogate could be attributed to relative preponderance of non–cancer-specific deaths in this patient population. These findings underscore the impact of varying the censoring criteria used in the definition of surrogate end points.
